# A Cross-Cultural Study of Filial Piety and Palliative Care Knowledge: Moderating Effect of Culture and Universality of Filial Piety

**DOI:** 10.3389/fpsyg.2021.787724

**Published:** 2021-12-03

**Authors:** Wendy Wen Li, Smita Singh, C. Keerthigha

**Affiliations:** ^1^College of Healthcare Sciences, James Cook University, Townsville, QLD, Australia; ^2^School of Social and Health Sciences, James Cook University, Singapore, Singapore

**Keywords:** palliative care knowledge, filial piety, filial obligation, universal psychological construct, contextualized personality construct, measurement invariance, moderation, PROCESS

## Abstract

Filial piety is a Confucian concept derived from Chinese culture, which advocates a set of moral norms, values, and practices of respect and caring for one’s parents. According to the dual-factor model of filial piety, reciprocal and authoritarian filial piety are two dimensions of filial piety. Reciprocal filial piety is concerned with sincere affection toward one’s parent and a longstanding positive parent-child relationship, while authoritarian filial piety is about obedience to social obligations to one’s parent, often by suppressing one’s own wishes to conform the demands of the parent. The primary aim of this study is to investigate the moderating effect of culture on the relationships between filial piety and palliative care knowledge. The secondary aim is to investigate whether filial piety is a universal construct across Singaporean and Australian cultures. A total of 508 participants living in Singapore and Australia were surveyed between May and October 2020. The final sample comprised of 406 participants, with 224 Singaporeans and 182 Australians. There were 289 females (71.1%), 115 males (28.3%), and two unspecified gender (0.6%) in the sample, with an average age of 27.27 years (SD = 9.79, range = 18–73). Results indicated a significant effect of culture on authoritarian filial piety and palliative care knowledge. Singaporeans showed higher authoritarian filial piety and higher palliative care knowledge than Australians. However, no effect of culture was found on reciprocal filial piety. Overall, no significant correlation existed between palliative care knowledge and reciprocal filial piety and authoritarian filial piety. For Singaporeans, a weak negative correlation was found between palliative care knowledge and authoritarian filial piety. In contrast, Australians and Singaporeans indicated a positive, moderate correlation between reciprocal and authoritarian filial piety. Further, culture moderated the relationship between authoritarian filial piety and palliative care knowledge. High authoritarian filial piety was associated with increased palliative care knowledge among Australians, while high authoritarian filial piety was associated with decreased palliative care knowledge among Singaporeans. The results support the conceptualization of filial piety as a possible psychological universal construct. In addition, the results point out an important implication that public health programs should target the appropriate filial piety types to enhance palliative care knowledge among Singaporeans and Australians.

## Introduction

### Background

According to World Health Organization [WHO] (2018), palliative care is person- and family-centered care that focuses on the improvement of the quality of life for both patients with a life-threatening illness and their families, through preventing and relieving suffering by the early identification, correct assessment, and treatment of pain and other physical, psychosocial, or spiritual problems. The need for palliative care is expanding due to the population aging and the growth in the prevalence of cancer and other chronic non-communicable diseases worldwide. Many people with life threatening illnesses hope for a good death with dignity (Cao et al., 2020), namely, dying with dignity that emphasizes on maintaining people’s autonomy at the end of their life (Chochinov, 2006). The provision of high-quality palliative care is a way to improve the quality of death, and many countries have continuously made progress in improving affordable access to palliative care (The Economist Intelligence Unit [EIU], 2015), including Australia and Singapore. The 2015 Quality of Death Index (The Economist Intelligence Unit [EIU], 2015), which ranks palliative care worldwide, revealed that Australia and Singapore are at the top of the Index at positions two and 12, respectively. These two countries are coping with a rapidly aging population. Hence, providing high-quality palliative care to ensure that people have a good life right until the end of their lives has risen in the agenda for healthcare policymaking. Since the outbreak of COVID-19, Australia and Singapore have also considered palliative care as an essential component of the public health response to the COVID-19 pandemic and include palliative care in their COVID-19 resolution (Krishna et al., 2020; Australian Institute of Health and Welfare [AIHW], 2021).

Although the 2015 Quality of Death Index indicates that both Australia and Singapore provide world-class palliative care services, palliative care services remain underutilized. For example, among 83,430 palliative care-related hospitalizations reported from public acute and private hospitals in Australia in 2018–2019, 57.3% opted and received palliative care, while 42.7% opted for and received other end-of-life care where the principal clinical strategy of care is not palliation (Australian Institute of Health and Welfare [AIHW], 2021). In Singapore, the utilization rate for in-patient palliative care was around 15% of all cancer deaths (which is the top cause of death in Singapore) in the financial year of 2009–2010 (Lien Centre for Palliative Care, 2011). These utilization rates suggest that palliative care utilization is not optimized and that more patients may be being cared for treatments than necessary.

A recent systematic review on palliative care education (Li et al., 2021a) suggests that barriers to utilizing palliative care services include patients’ and families’ misunderstandings and lack of knowledge of palliative care; reluctance and fear to utilize palliative care; and ignorance and lack of awareness of resources in relation to palliative care. Programs that aim to improve palliative care knowledge are primarily targeted to healthcare professionals, followed by family caregivers. Community engagement in the improvement of palliative care knowledge is limited. The authors recommend that community engagement in improving palliative care knowledge will bolster the credibility of palliative care as a ‘public health issue,’ which will help develop stronger palliative care policy and boost palliative care utilization. The higher palliative care utilization rate in Australia compared to Singapore warrants a comparative investigation of palliative care knowledge in the two countries.

One of the goals of palliative care is to help patients and their families make medically important decisions (Rome et al., 2011). Family members play a vital role in the palliative care decision-making process. When the patient of palliative care is the parent, a filial obligation is an important factor in the patient’s childrens’ decision-making within the context in which children occupy two different roles—being children and caregivers. Although filial piety is a Confucian concept derived from Chinese culture, filial piety is used to connote the concept of filial obligation in many Asian cultures with their own indigenous terms in the cultures such as in the three dominant cultures in Singapore: the Chinese, Indian and Malay.

In Chinese culture, filial piety has long been studied as a set of cultural and moral norms, values, and practices of respect and caring for one’s parents. In the Chinese language, filial piety translates to *Xiao*. Ideographically, the Chinese character of *Xiao* is comprised of two other characters with the *old* on the top and the *son* at the bottom, written from the top to the bottom. This ideograph indicates the hierarchical structure of the family and the responsibility that the young is expected to support the old in the family (Ikels, 2004). Hence, filial piety in Chinese culture not only requires filial duties (e.g., material support and co-residence with aging parents) from the children to their (grand)parents but also obedience to (grand)parental demands (Ho, 1996; Yeh, 2003; Li et al., 2020b). This child-parent relationship places filial piety at the core of the Chinese familial ideology and moral worldview (Hwang, 1999; Yeh and Bedford, 2003; Bedford and Yeh, 2019; Li and He, 2019). Filial piety is thus a framework and the starting point of the discussion of aged care in Chinese culture (Li et al., 2010).

In Indian culture, the parent-child relationship in Hindu ideology is built upon the concept of filial devotion and love of Shravan Kumar, who fulfills all the desires and wishes of his parents (Rao et al., 2003). Akin to this is the notion of *seva* in Indian culture (Sharma and Kemp, 2012). *Seva* is the Indian word equivalent to the Chinese *Xiao*, which refers to “long-term bonds of intergenerational reciprocity and affection, in which juniors provide care for their senior parents in old age and after death, as ancestors in return for all of the effort, expense and love their parents expended to producing raise them in infancy and childhood” (Lamb, 2002, p. 304). *Seva* is central in the Indian parent-child relationship, requiring support and care from the child to the (grand)parent and the filial duties of a son for his parents (Tiwari and Pandey, 2013). Similar to *Xiao* in Chinese culture, a key dimension of *seva* is multi-generational co-residence (Sharma and Kemp, 2012), namely joint family (Tiwari and Pandey, 2013), reflecting interdependence and the collectivist nature of Indian culture. In the joint family, aging parents are more likely to be cared for by their adult children as opposed to formal support sources (Sharma and Kemp, 2012). In that regard, *seva* is a natural backdrop for the discussion of aged care in Indian culture.

In Malay culture, the indigenous term of filial piety is *Ketaatan Kepada Ibu Bapa* (Tan et al., 2019), meaning loyalty toward the parents. Scholars suggest that *Ketaatan Kepada Ibu Bapa* in Malaysia is close to their traditional Confucian origins and promotes accentuate obedience (Thomas, 1990). Research has found that Malay children internalize *Ketaatan Kepada Ibu Bapa* not only from their parents but also from mass media, and their relatives, friends, and teachers (Ismail et al., 2009). A study investigating filial responsibility among Malaysian university students found that the Malay students had slightly higher mean scores in filial responsibility than their Chinese and Indian counterparts (Tien et al., 2009).

From a collective perspective, in addition to the cultural underpinnings of each of the ethnic groups, the Singapore government endorses Asian ideologies of family-based care for elders. The principles are popularly encouraged and promoted as virtues that bind society together. For example, “Family is the basic unit of society” is one of the four norms in the *Shared Values White Paper of 1991* (Prime Minister’s Office, 1991). Hence, the importance of filial duties is ingrained in Singapore society.

Although there is no corresponding concept of filial piety, there is a concept of filial obligation in Western culture. Filial obligations refer to children’s special obligations toward their parents, particularly adult children toward their elderly and needy parents (Schinkel, 2012). Western filial obligations are built upon responsibility, respect, and care that reflect the appreciation for past parental sacrifices, the special nature of the parent-child relationship, and the moral responsibilities to the elderly (Jones et al., 2021). Thus, even if the centrality of family relationships in the Western culture is the husband-wife relationship and not the parent-children relationship as in Chinese culture (Li, 2013), Western adult children fulfill their filial obligations to their parents by offering material support to, and even co-reside with, their parents. With the aging-in-place policy that promotes the practice where older people continue to live in their own homes as long as possible (Li, 2013), many Western countries have shifted from institutional care to formal or informal homecare (Schinkel, 2012). The success of the aging-in-place approach increasingly relies on filial obligations in aged care in Western culture. In Australia, the family is regarded as a palliative home care unit (Kirby et al., 2018). Family caregivers provide major assistance with palliative care provision in the home, including symptom evaluation and management, administration of medication, and hygiene and daily care (Hudson et al., 2011). This palliative home care for the elderly is often provided by the patient’s adult children who fulfill their filial obligations.

It is pertinent to point out that in liberal Western societies, caring for their young children is a parental obligation and morally and legally required, while the filial obligation to look after aged parents is voluntary and not morally required (Fan, 2006). In this regard, filial responsibility in Western cultures is fundamentally different from filial piety in the three dominant cultures in Singapore, where filial piety is deemed a moral norm and ideology. An empirical investigation into differences in filial obligation (or filial piety) between Singaporean and Australian cultures is warranted.

Moreover, considering the role that filial obligation plays when children are involved in making decisions on palliative care for their parents who suffer life-threatening illness, and that palliative care knowledge is a barrier for the utilization of palliative care services, it is worth exploring to what extent filial piety is related to palliative care knowledge. As suggested by Li (2013), a filial child is expected to serve his/her parent with medicine on the parent’s deathbed. Consequently, not providing the seriously sick parent with curative treatment is regarded as a failure of fulfilling the moral duty of an adult child. Sending the sick parent to palliative care is viewed as an abandonment of the ill parent and socially unaccepted. Filial piety thus may be an underlying reason that prevents Singaporeans from obtaining palliative care knowledge and utilizing hospice and palliative care services. Contrary to Singaporean culture, Australians may believe that providing an ill parent with palliative care is their filial obligation which can improve the quality of life and offer a good death at the very end. This consideration may motivate Australians to obtain palliative care knowledge and utilize palliative care services. Therefore, the cultural background may impact the relationship between filial piety and palliative care knowledge, which warrants an empirical investigation. To the authors’ knowledge, there are no studies investigating the relationship between filial piety and palliative care knowledge or if this relationship is moderated by culture.

Based on the phenomenon that the manifestation of filial piety may differ by culture but filial piety-based interaction between children and parents exists in all cultures, recent research into the psychology of filial piety proposes that filial piety can be studied as a contextualized personality construct that functions as a universal human motivation with cross-cultural generalizability (Bedford and Yeh, 2019). As such, the overlap among Chinese *Xiao*, Indian *seva*, Malaysian *Ketaatan Kepada Ibu Bapa* and Western filial obligations and their shared implications for aged care offer a cross-cultural foundation for the new research trend that investigates if filial piety can be re-conceptualized as a universal construct across cultures. This re-conceptualization of filial piety marks a departure of the psychology of filial piety from solely focusing on Chinese culture-specific norms.

Bedford and Yeh (2019) proposed that filial piety can be studied as a contextualized personality construct based on their dual filial piety model. The dual-factor model of filial piety theorizes that filial piety is comprised of two dimensions: reciprocity and authoritarianism (Yeh, 2003; Yeh and Bedford, 2003). Reciprocal filial piety refers to sincere affection toward one’s parent, which has grown from a longstanding positive parent-child relationship. Individuals with attitudes of reciprocal filial piety care about their parents out of sincerity and gratitude. Authoritarian filial piety is concerned with obedience to social obligations to one’s parent, often by suppressing one’s own wishes to conform to the demands of the parent (Yeh, 2009; Bedford and Yeh, 2019). Reciprocal filial piety is driven by the relational need for social connections, whereas authoritarian filial piety is stimulated by the psychological need for collective identities (Hwang, 1999; Chen et al., 2016). Reciprocal and authoritarian filial piety does not mutually exclude one another. Instead, they are intertwined and function simultaneously in different degrees, depending on the situation. For example, they may function together to achieve the same outcome because they both endorse intergenerational support. The intergenerational support by reciprocal filial piety is offered through accumulated affection. In contrast, the intergenerational support by authoritarian filial piety is achieved *via* regulating behavior so as to meet the minimum social expectations for adult children supporting their parents (Bedford and Yeh, 2019). The dual dimensions of filial piety reflect the notion of contextualized personality which purports that personality manifests in different ways (e.g., through accumulated affection or regulating behavior) across various social roles and contexts where people inhabit throughout their lives (Dunlop, 2015).

Contextualized personality refers to a stable set of tendencies and characteristics of thought, feelings, and behaviors that repeatedly appear within a given context (Heller et al., 2007). The primary idea behind this concept is that while personality characteristics are inclined to be stable within a specific context, they may differ considerably across different social roles and contexts because of psychological prompts or demands that are unique to certain contexts (Dunlop, 2015; Fisher et al., 2017). As such, personality characteristics are manifested within the recurring social roles and contexts (Dunlop and Hanley, 2019). Consequently, a person may display a certain personality characteristic within one context and a completely opposite characteristic within another context, dependent on varying contextual circumstances that bring about different response tendencies (Fisher et al., 2017). For example, when children experience the decision-making process of whether to utilize palliative care services for their seriously sick parent, the role-identities of children and caregivers that are both relevant to the decision are activated in cognition. However, at a certain moment, only one subset of these role-identities is more accessible. The activated and accessible role-identity and its related personality characteristics of filial piety, in turn, are likely to influence their decision making (Heller et al., 2009) and motivate them to utilize or to avoid palliative care services. The contextualized personality to filial piety moves filial piety research beyond the focus on cultural and social norms to the generalizability of filial piety across cultures. This novel conceptualization warrants an empirical investigation on whether filial piety is a universal construct in cultural contexts outside Chinese culture.

### The Present Study

**FIGURE 1:**
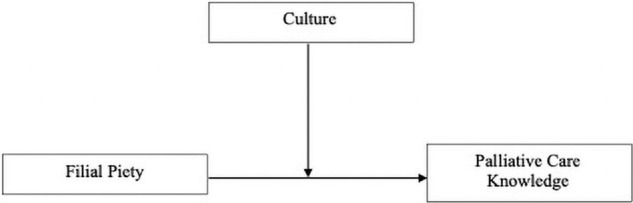
Conceptual model of the mediation analyses.

The primary aim of the present study is to investigate the moderating effect of culture on the relationships between filial piety and palliative care knowledge in a sample consisting of Singaporeans and Australians. The secondary aim is to assess whether filial piety is a universal construct across Singaporean and Australian cultures. Due to the exploratory nature of the current study, research questions, rather than *priori* hypotheses, are proposed. To achieve the primary research aim, four research questions are investigated, with Figure 1 presenting the conceptual model that shows the moderation pathway:

RQ1. Are there differences in reciprocal and authoritarian filial piety between Singaporean and Australian cultures?

RQ2. Are there differences in palliative care knowledge in Singaporean and Australian cultures?

RQ3. Are reciprocal and/or authoritarian filial piety correlated to palliative care knowledge?

RQ4. Does culture moderate the relationship between filial piety and palliative care knowledge?

The secondary research aim is not directly tested. Rather, this question will be answered by the results of the relationships between filial piety and palliative care knowledge across the two cultural groups. According to Norenzayan and Heine (2005), there are three categories of psychological universals—the accessibility universal, functional universal, and existential universal. A three-step approach can be employed to identify if a psychological construct is universal. The first step is to examine if the construct is an accessibility universal. A construct is an accessibility universal if it is cognitively available to most people in most cultures, it has the same use in all cultures, and it is accessible to the same degree across cultures. When a psychological construct shows cultural variability in accessibility, the second step is to assess whether it is a functional universal. A psychological construct is a functional universal if the pattern of the relationship between the construct and other variables (e.g., palliative care knowledge in the present study) is the same across the cultures being studied, even though the strength of the pattern differs. When a psychological phenomenon demonstrates cultural variability in the functional dimension, the third step is to identify if it is an existential universal. A psychological construct is an existential universal if it is cognitively available to normal adults across cultures, even if the ways or frequency of using the construct may vary distinctly across the cultures. The current study follows this approach to examine whether filial piety is a universal psychological construct that can be generalized in Singaporean and Australian cultures.

## Materials and Methods

### Participants

A total of 508 participants took part in the survey. They were categorized according to their country of residence: Singapore or Australia. Participants who lived outside Singapore and Australia (*N* = 45) were removed. To minimize intra-group variation, non-Asians in the Singapore sample (*N* = 9) and Asians in the Australian sample (*N* = 14) were removed. Further data cleaning (see the section “Data Cleaning”) resulted in a final N of 406 with an age range of 18–73 years (*M* = 27.27, *SD* = 9.79). The overall sample consisted of 224 Singaporeans (*M*_*age*_ = 26.72, *SD* = 7.8) and 182 Australians (*M*_*age*_ = 27.94, *SD* = 11.77). The demographic characteristics of the final sample are reported in Table 1.

**TABLE 1:** Demographic characteristics of the participants.

Variables	Overall	Singapore	Australia
	**M (SD)**	**M (SD)**	**M (SD)**
**Age**	**27.27 (9.79)**	**26.72 (7.8)**	**27.94 (11.77)**
	**% (N)**	**% (N)**	**% (N)**

Total participants	100 (406)	55.2 (224)	44.8 (182)
Ethnicity			
Chinese		51.8 (116)	
Indian		44.6 (100)	
Malay		3.6 (8)	
European australian			87.9 (160)
Indigenous australian			12.1 (22)
Gender			
Male	28.3 (115)	27.2 (61)	29.5 (54)
Female	71.1 (289)	72.3 (162)	70.0 (127)
Unspecified/intersex	0.6 (2)	0.5 (1)	0.5 (1)
Highest level of formal education			
High school/certificate/diploma	14.5 (59)	13 (29)	16.5 (30)
Undergraduate	79 (320)	76.3 (171)	81.9 (149)
Postgraduate	6.5 (27)	10.7 (24)	1.6 (3)
Occupation			
Student	54.8 (212)	56.7 (118)	52.5 (94)
Healthcare services	10.6 (41)	9.7 (20)	11.7 (21)
Education	9.8 (38)	12.5 (26)	6.7 (12)
Finance sector	0.7 (2)	0.5 (1)	0.5 (1)
Hospitality/retail	4.1 (16)	2.4 (5)	6.1 (11)
Public servant	3.3 (13)	2.4 (5)	4.5 (8)
Social services/community organization	3.6 (14)	2.4 (5)	5.2 (9)
Trade-person	1.5 (6)	0.9 (2)	2.2 (4)
Other	11.6 (45)	12.5 (26)	10.6 (19)
Missing data	4.7 (19)	7.1 (16)	1.6 (3)
Annual income			
0–$18,200	55.6 (225)	61 (136)	49.2 (89)
18,201–$37,000	16.6 (67)	14 (31)	19.9 (36)
37,001–$80,000	18.6 (75)	15.7 (35)	22.1 (40)
80,001–$120,000	7.2 (29)	7.1 (16)	7.2 (13)
More than $120,001	2.0 (8)	2.2 (5)	1.6 (3)
Missing data	0.5 (2)	0.5 (1)	0.5 (1)
Marital status			
Single	68.6 (277)	79.5 (178)	55 (99)
Divorces/widowed/separated	4.2 (17)	0.5 (1)	8.9 (16)
Married/*de facto* relationship	27.2 (110)	20 (45)	36.1 (65)
Missing data	0.5 (2)	0 (0)	0 (0)

### Measures

#### Demographic Variables

Several demographic variables were measured including age, gender, country of residence, highest level of education, marital status, occupation, and income.

#### Filial Piety

Filial piety was measured by the standardized dual filial piety scale (Yeh and Bedford, 2003). The 16-item scale produces totals for reciprocal and authoritarian filial piety (eight items for each subscale) using a 6-point scale in which 1 = “*Extremely unimportant”* and 6 = “*Extremely important.”* Sample items for reciprocal filial piety include “Be concerned about my parents’ health” and “Support my parents’ livelihood to make their lives more comfortable.” Sample items for authoritarian filial piety include “Have at least one son for the succession of the family name” and “Give up my aspirations to meet my parents’ expectations.” Higher scores indicate greater reciprocal and authoritarian filial piety. The scale has previously demonstrated good internal consistency with Cronbach’s alphas of0.92 and0.86 for reciprocal filial piety and authoritarian filial piety, respectively (Li et al., 2021b). In this study, Cronbach’s alphas for reciprocal filial piety and authoritarian filial piety were0.86 and0.82, respectively. The reliability analyses of the scale in the current study are presented in the section “Reliability Analysis” after measurement invariance was tested.

#### Palliative Care Knowledge

Palliative care knowledge was assessed using the palliative care knowledge scale (Kozlov et al., 2017). This is a self-report questionnaire with 13 dichotomous questions (true = 1, false = 0) about various domains of palliative care knowledge. Six reverse items were re-coded. Higher scores indicate higher levels of palliative care knowledge. Example items for the scale include, “A goal of palliative care is to address any psychological issues brought up by serious illness” and “Palliative care helps the whole family cope with a serious illness.” The scale has fair reliability with the Kuder-Richardson 20 coefficient (KR-20) of0.71 by the scale author (Kozlov, 2016). The reliability analysis of the scale in the current study is presented in the section “Reliability Analysis”.

### Procedure

Ethical clearance for the study was granted by the Human Research Ethics Committee James Cook University, Australia (Ref. H8021). Research posters carrying the link of the survey was circulated *via* the University’s research sites (Sona Systems^®^) and social media platforms (e. g., Facebook). Interested participants would respond to the link, which directed them to the Qualtrics survey site. After reading the preliminary information about the survey, they had an option to proceed or exit the study. Psychology undergraduate participants of James Cook University received course credits for their research participation. This online cross-sectional survey was administrated between May and October 2020. The survey took about 20 minutes to complete.

### Data Cleaning

Thirty-two incomplete surveys were removed. One participant was removed due to unengaged responding (i.e., answers to all questions were the highest possible value). Using boxplots, five extreme univariate outliers (four on palliative care knowledge and one on reciprocal filial piety), which scored three box lengths above or below the box boundary, were identified and winsorized to match the next highest/lowest non-outlying values. One multivariate outlier was detected using Mahalanobis distance figures (using a criterion α = 0.001, *df* = 5 [equal to the number of variables], critical χ^2^ = 20.515; Tabachnick and Fidell, 2013, p.99) and removed, resulting in 406 participants for the final analysis.

Missing data were minimal (0.2–0.8%) with 12 items missing one or two values across the three measures. Little’s missing completely at random (MCAR) test were conducted. The results indicated that data were missing completely at random for the filial piety scale (χ^2^ = 100.79, *df* = 90, *p* = 0.21) and palliative care knowledge (χ^2^ = 64.62, *df* = 48, *p* = 0.06). Expectation-maximization (EM) algorithm, an approach to maximum-likelihood based missing data method (Dong and Peng, 2013), was used to replaced missing data. Maximum likelihood estimation and EM were chosen in the consideration that, firstly, maximum likelihood estimation is consistent with its use in the multigroup confirmatory factor analysis employed in the measurement invariance test in the current study (see the section Test of Measurement Invariance of the Dual FilialPiety Scale); and secondly, EM estimation is an unbiased and efficient missing data replacement method when the missingness is ignorable (Dong and Peng, 2013). The ignorable missingness is indicated by the small size of missing data and MCAR (Graham, 2009). The missing data in the current study met the ignorability criteria.

The Kolmogorov-Smirnov test was used to assess the normality of the scales. All scales violated the assumption of normality (*p* < 0.001). Levene’s test indicated that the palliative care knowledge scale violated the homogeneity assumption, *p* = 0.009. In addition, the transformations of variables failed to adequately adjust for the normal distribution using log and square root. Bootstrapping with 5,000 iterations was thus employed in the analysis (Field, 2017; Li et al., 2020a).

### Data Analysis

Using G^∗^Power version 3.1.9.4 (Faul et al., 2007), a *post hoc* power analysis was performed to test whether to examine whether the sample size of 406 was adequate to conduct multiple linear regression. The analysis revealed a statistical power level of 0.99 with a medium effect size of 0.15, an alpha level of 0.05 and a predictor number of 4 (one independent variable, one moderator, and two covariates).

To determine the measurement equivalence across the two cultural groups, measurement invariance was tested using IBM SPSS Amos Graphics version 27. Measurement invariance test using multigroup confirmatory factor analysis (MGCFA) involves a set of sequential models that are conducted in a logically ordered and increasingly restrictive fashion with a four-step process (Byrne, 2004; Byrne and Van de Vijver, 2010; Boer et al., 2018; Miller et al., 2019). First, a baseline model is established for testing configural invariance in which items show the same configuration loadings in each cultural group. Good model fit of each baseline model suggests that the item factor structure is similar across groups. Second, if configural invariance is satisfied, the metric invariance model in which factor loadings are constrained to be equal across cultural groups is tested against the configural model. Metric invariance is met if the model fit of the metric invariance model is not significantly different from the configural model. Third, if metric invariance is supported, the scalar invariance model where all factor loadings and item intercepts are constrained to be equal across groups is tested against the metric invariance model. Scalar invariance is assumed if the model fit of the scalar invariance model does not significantly differ from the metric invariance model. Fourth, if scalar invariance is demonstrated, the residuals/measurement errors invariance model where residuals/measurement errors are constrained to be equal across groups is tested against the scalar model. Residuals/measurement errors invariance is supported if the residuals/measurement errors invariance model is not statistically different from the scalar model (Byrne, 2004; Kline, 2016; Boer et al., 2018).

To examine model fit, chi-square goodness-of-fit statistics (χ^2^) and model fit indexes of Tucker-Lewis index (TLI), comparative fit index (CFI), root mean square error of approximation (RMSEA), and the standardized root mean-square residual (SRMR) were used. If χ^2^ value is large and significant, the model is considered an inadequate fit. Considering that χ^2^ is highly dependent on sample size (Hu and Bentler, 1999), the ratio of χ^2^ to degrees of freedom (PCMIN/DF) is used to evaluate model fit. PCMIN/DF less than 3 is desired for a good model fit (Tabachnick and Fidell, 2013). TLI and CFI greater than0.95 is considered a good model fit (Hu and Bentler, 1999), and greater than0.90 are acceptable for adequate fits (Byrne, 2016; Kline, 2016). RMSEA less than0.05 indicates a good model fit (Hu and Bentler, 1999), and close to0.08 suggests an adequate model fit (Byrne, 2016; Kline, 2016). SRMR less than0.08 is considered a good model fit (Hu and Bentler, 1999), and less than0.10 indicates a mediocre fit (Byrne, 2016; Kline, 2016).

Data analysis for testing the research questions (RQ) was performed using IBM’s SPSS version 27. An independent samples *t-*test was employed to determine whether there were differences in reciprocal and authoritarian filial piety, and palliative care knowledge between the two cultural groups (RQ1 and RQ2). To assess the size and direction of the linear relationship between reciprocal filial piety, authoritarian filial piety, and palliative care knowledge in the overall sample, a bivariate Pearson’s product-moment correlation coefficient (*r*) was conducted (RQ3). Model 1 of PROCESS v3.5.3 macro for SPSS was used for moderation analysis (consisting of variables culture, reciprocal/authoritarian filial piety, and the interaction effect between culture and reciprocal/authoritarian filial piety; RQ4) with 5000 resamples to bootstrap 95% confidence intervals (Hayes, 2018; Li and Xie, 2020; Xie et al., 2021).

To control the covariates in the moderation models, independent samples of *t*-test and chi-square test were performed to determine which demographic factors of gender, age, education, marital status, employment, and annual income had statistical differences in all variables under investigation. *T*-test indicated that the cultures did not differ in terms of age, *t*(404) = −1.25, *p* = 0.21, with the Singaporean participants (*M* = 26.72, *SD* = 7.80) being slightly younger than the Australian participants (*M* = 27.94, *SD* = 11.76). The chi-square tests showed that the two cultures significantly differed in terms of highest level of education, χ^2^(2, *N* = 406) = 13.66, *p* < 0.001, Cramer’s *V* = 0.18 and marital status, χ2 (2, *N* = 404) = 35.03, *p* < 0.001, Cramer’s *V* = 0.29. However, the two cultures did not significantly differ on gender, χ^2^(2, *N* = 406) = 3.23, *p* = 0.85, Cramer’s *V* = 0.03; employment, χ^2^(8, *N* = 387) = 11.63, *p* = 0.16, Cramer’s *V* = 0.17; and annual income, χ^2^(4, *N* = 404) = 7.04, *p* = 0.13, Cramer’s *V* = 0.13. Consequently, education and marital status were entered into the model as covariates.

## Results

### Preliminary Analysis

#### Test of Measurement Invariance of the Dual Filial Piety Scale

The first step was to establish the baseline model, based on which the configural model was tested. The baseline model was established by using confirmatory factor analysis for evidence of model fit of the 16-item filial piety scale and tested separately in Singaporean and Australian participants. The hypothesized model for the Singaporean participants generated an adequate model according to the fit indices: χ^2^(103) = 233.853, *p* < 0.001, PCMIN/DF = 2.270, CFI = 0.907, TLI = 0.891, RMSEA = 0.075, 90% CI [0.071, 0.088], SRMR = 0.087; while the hypothesized model for the Australian participants was found to be of poor fit: χ^2^(103) = 251.390, *p* < 0.001, PCMIN/DF = 2.441, CFI = 0.851, TLI = 0.826, RMSEA = 0.089, 90% CI [0.075, 0.103], SRMR = 0.095. Inspection of the modification indices showed moderate values of error covariances between items 1 and 5 (MI = 18.971, PCS = 0.115) and between items 4 and 16 (MI = 19.107, PCS = 0.214). Scrutiny of the content for each of these items revealed evidence of considerable overlap (e.g., using similar wordings and/or meaning of the items appearing similar to each other) between each of these item pairs; for example, “Be concerned about my parents’ health” (item 1) vs. “Be concerned about my parents’ general well-being” (item 5) and “Let my income be handled by my parents before marriage” (Item 4) vs. “Live with my parents (or parents-in-law) when married” (Item 16). The overlap of these items can trigger error covariances (Byrne, 2004). Considering this applicable modification, these error terms of items 1 and 5 and of items 4 and 16 were subsequently covaried as free parameters in the model for each group (Byrne and Van de Vijver, 2010; Kline, 2016). The modified Australian baseline model generated an acceptable model: χ^2^(101) = 208.383, *p* < 0.001, PCMIN/DF = 2.063, CFI = 0.892, TLI = 0.868, RMSEA = 0.077, 90% CI [0.062, 0.092], SRMR = 0.089. The modified Singaporean baseline model resulted in better model fits: χ^2^(101) = 222.595, *p* < 0.001, PCMIN/DF = 2.270, CFI = 0.913, TLI = 0.897, RMSEA = 0.073, 90% CI [0.060, 0.087], SRMR = 0.086.

Following the establishment of the modified baseline model, the configural model (model 1) in which the basic factors of the baseline model were constrained to equality across the two cultural groups was tested. In this model, the same parameters that had been estimated in the modified baseline model for each group separately were estimated simultaneously using the multigroup modeling (Byrne, 2016). The model fit indices reported acceptable model fit: χ^2^(202) = 430.978, *p* < 0.001, PCMIN/DF = 2.134, CFI = 0.905, TLI = 0.887, RMSEA = 0.053, 90% CI [0.046, 0.060], SRMR = 0.086, supporting that the overall pattern of parameters was equal across the Singapore and Australia groups, namely, the configural invariance was supported.

Second, the metric invariance was tested. The model (Model 2), where all factor loadings and two error covariances (Items 1 and 5; Items 4 and 16) (Byrne, 2016) were constrained to be equal across the Singaporean and Australian groups, was tested against the configural model. The results showed adequate model fits: χ^2^(216) = 460.383, *p* < 0.001, PCMIN/DF = 2.131, CFI = 0.898, TLI = 0.887, RMSEA = 0.053, 90% CI [0.044, 0.052], SRMR = 0.088. The comparison between Model 1 and Model 2 yielded the following values: Δχ^2^(14) = 29.405, *p* < 0.05 and ΔCFI = 0.007. Not surprisingly, the χ^2^ difference test showed the evidence of non-invariance due to two error covariances being added to the model (Byrne, 2016). The CFI difference test indicated invariance because the ΔCFI value of 0.007 was less than the 0.010 cut-off point recommended by Cheung and Rensvold (2002). Considering that Δχ^2^ is sensitive to the sample size (Cheung and Rensvold, 2002; Meade et al., 2008) and characterizes exact measurement invariance while the tested models at the best is only approximate invariance (Byrne, 2016), Meade et al. (2008) suggested that if ΔCFI argues for invariance and the sample size is greater than 200 (which is the case of the present study), although Δχ^2^ is significant, the differences of the measurement between groups are possibly insignificant and further analyses could progress. Hence, metric invariance across the Singaporean and Australian groups was established.

Third, the scalar invariance (Model 3), in which all factor loadings, two error covariances and item intercepts were constrained equal across two groups (Byrne, 2004), was tested against the metric invariance model. Results showed poor model fits: χ^2^(230) = 690.069, *p* < 0.001, PCMIN/DF = 2.957, CFI = 0.809, TLI = 0.803, RMSEA = 0.070, 90% CI [0.064, 0.076], SRMR = 0.090. The comparison between Model 2 and Model 3 produced the following values: Δχ^2^(14) = 229.686, *p* < 0.001 and ΔCFI = 0.089. The results from Δχ^2^ and ΔCFI (>0.010) indicated that full scalar invariance was not supported. To test partial scalar invariance, the intercept restriction for items 2, 3, 9, 10, 14, and 16 were released based on the intercept differences between the two cultural groups in the modification indices. The modified model (partial scalar invariance model; Model 3b) demonstrated an improvement of model fit: χ^2^(224) = 477.849, *p* < 0.001, PCMIN/DF = 2.133, CFI = 0.894, TLI = 0.887, RMSEA = 0.053, 90% CI [0.047, 0.060], SRMR = 0.090. The comparison between Model 2 and Model 3b indicated support to partial scalar invariance with ΔCFI = 0.004, suggesting that item intercepts were partially equivalent across the two cultural groups. The fit indices are summarized in Table 2. As a result of the rejection of full scalar invariance, the fourth step of testing the residuals/measurement errors invariance was not proceeded.

**TABLE 2:** Fit indices for the filial piety scale models.

	χ^2^(*df*)	CFI	TLI	SRMR	RMSEA [90% CI]	Model (M) comparison	Δχ^2^(*df*)	ΔCFI
Baseline model								
Singapore	222.595 (101)***	0.913	0.897	0.086	0.073 [0.060, 0.087]	–	–	–
Australia	208.383 (101)***	0.892	0.868	0.089	0.077 [0.062, 0.092]	–	–	–
M1: configural	430.978 (202)***	0.905	0.887	0.086	0.053 [0.046, 0.060]	–	–	–
M2: metric	460.383 (216)***	0.898	0.887	0.088	0.053 [0.044, 0.052]	2 vs.1	29.405 (14)*	0.007
M3: scalar	690.069 (230)***	0.809	0.803	0.090	0.070 [0.064, 0.076]	3 vs. 2	229.686 (14)***	0.089
M3b: partial scalar	477.849 (224)***	0.894	0.887	0.090	0.053 [0.047, 0.060]	3b vs. 2	17.466 (8)*	0.004

In the Baseline Model, error terms of items 1 and 5, and items 4 and 16 were covaried as free parameters in each group. In Model 3b, intercepts of items 2, 3, 9, 10, 14, and 16 were freely estimated. *p < 0.05; ***p < 0.001.

#### Reliability Analysis

The intercorrelations, descriptives, and reliability coefficients of reciprocal filial piety, authoritarian filial piety, and palliative care knowledge in overall Singapore and Australia Samples are presented in Table 3. Both reciprocal and authoritarian filial piety scales demonstrated good reliability with alpha coefficients between0.82 and0.89 in the overall Singaporean and Australian samples. The KR-20s of the scale were between0.61 and0.69. According to Norman and Streiner (2008), a reliability coefficient above0.60 is acceptable for a health knowledge scale. The resulting scales thus showed acceptable levels of internal consistency.

**TABLE 3:** Intercorrelations, descriptives, and reliability coefficients of reciprocal filial piety, authoritarian filial piety, and palliative care knowledge in the overall, Singapore, and Australia samples.

Sample	Variable	1	2	3	*M*	*SD*	*Reliability coefficients*
Overall (*N* = 406)	(1) RFP	–	–	–	34.32	4.64	0.86
	(2) AFP	0.40**	–	–	20.59	6.02	0.82
	(3) PCK	0.11*	−0.05	–	11.30	1.92	0.66
Singapore (*N* = 224)	(1) RFP	–	–	–	34.44	4.81	0.89
	(2) AFP	0.39**	–	–	21.56	5.86	0.82
	(3) PCK	0.08	−0.24**	–	11.56	1.70	0.61
Australia (*N* = 182)	(1) RFP	–	–	–	34.17	4.42	0.84
	(2) AFP	0.40**	–	–	19.39	6.03	0.83
	(3) PCK	0.14	0.08	–	10.07	2.12	0.69

*p < 0.05; **p < 0.01; RFP, reciprocal filial piety; AFP, authoritarian filial piety; PCK, palliative care knowledge.

### Test of Research Questions

#### Test of Research Question 1

The independent samples *t-*test indicated no statistically significant effect of culture on reciprocal filial piety (*p* = 0.56). However, the effect of culture on authoritarian filial piety was statistically significant, *t*(404) = 3.67, *p* < 0.001, two-tailed, *d* = 0.37. Authoritarian filial piety was significantly higher in Singaporeans (*M* = 21.56, *SD* = 5.86) as compared to Australians (*M* = 19.39, *SD* = 6.03). Taken together, findings provided partial affirmation for RQ1.

#### Tests of Research Question 2

The independent samples *t-*test showed that the effect of culture on palliative care knowledge was statistically significant *t*(404) = 3.14, *p* = 0.002, two-tailed, *d* = 0.31. Palliative care knowledge was significantly higher in Singapore (*M* = 11.56, *SD* = 1.70) as compared to Australia (*M* = 10.97, *SD* = 2.12). Findings indicated that RQ2 was affirmed.

#### Test of Research Question 3

The bivariate Pearson’s product-moment correlation coefficient (*r*) showed that there was no statistically significant bivariate correlation between palliative care knowledge and authoritarian filial piety (*p* = 0.34). However, a positive and weak bivariate correlation was found between palliative care knowledge and reciprocal filial piety, *r* = 0.11, *p* < 0.05; and a positive and moderate bivariate correlation found between reciprocal filial piety and authoritarian filial piety, *r* = 0.40, *p* < 0.001. Results showed no statistically significant bivariate correlation between palliative care knowledge and reciprocal filial piety (*p* = 0.24) in the Singapore sample. However, there was a negative and weak bivariate correlation found between palliative care knowledge and authoritarian filial piety, *r* = −0.24, *p* < 0.001; and a positive and moderate bivariate correlation found between reciprocal filial piety and authoritarian filial piety, *r* = 0.39, *p* < 0.001.

In the Australia sample, results showed no statistically significant bivariate correlation between palliative care knowledge and reciprocal filial piety (*p* = 0.06); and palliative care knowledge and authoritarian filial piety (*p* = 0.30). However, a positive and moderate bivariate correlation was found between reciprocal filial piety and authoritarian filial piety, *r* = 0.40, *p* < 0.001.

The intercorrelations and descriptive statistics of reciprocal filial piety, authoritarian filial piety, and palliative care knowledge in the overall Singapore, and Australia samples are summarized in Table 3. Taken together, findings provided partial affirmation for RQ3.

#### Test of Research Question 4

**FIGURE 2:**
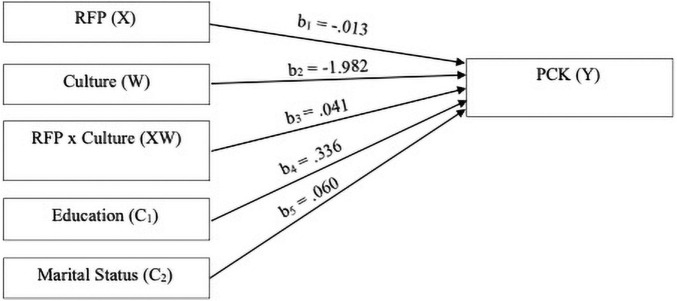
Main and interaction effects of reciprocal filial piety (RFP) and culture on palliative care knowledge (PCK).

**FIGURE 3:**
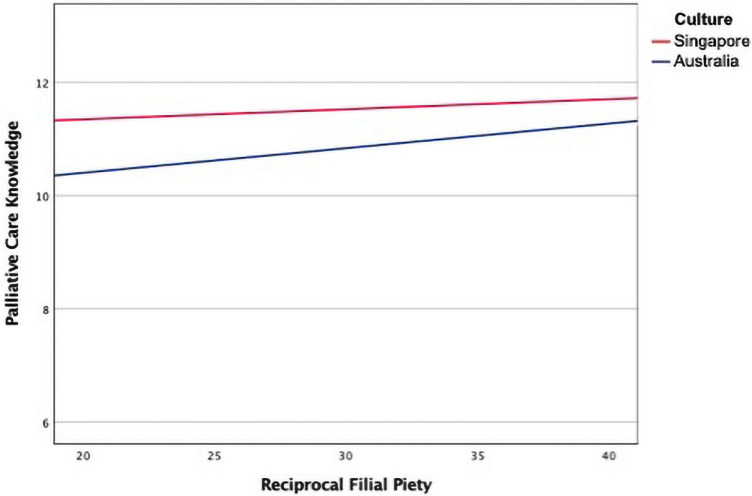
The moderating effect of culture on the relationship between reciprocal filial piety and palliative care knowledge.

The moderation analysis indicated that the interaction effect between culture and reciprocal filial piety was not found to significantly predict palliative care knowledge whilst controlling for education and marital status in the model, *F*(1, 398) = 0.98, *p* = 0.32, bias-corrected 95% CI [−0.040, 0.123]. Therefore, culture is not a significant moderator in reciprocal filial piety’s effect on palliative care knowledge. Table 4 and Figures 2, 3 present the regression parameter estimates, statistical diagram, and a visual representation of the moderation model, respectively.

**TABLE 4:** Summary of regression analysis examining the moderating effect of culture on the relationship between reciprocal filial piety and palliative care knowledge.

		Coeff.	*SE*	*t*	*p*
Constant	*i*_*Y*_	11.834	2.173	5.446	<0.001
Reciprocal filial piety (*X*)	*b*_1_	−0.013	0.062	−0.212	0.832
Culture (*W*)	*b*_2_	−1.982	1.435	−1.381	0.168
Reciprocal filial piety × culture (*XW*)	*b*_3_	0.041	0.041	0.991	0.322
Education (*C*_1_)	*b*_4_	0.336	0.211	1.595	0.112
Marital status (*C*_2_)	*b*_5_	0.060	0.110	0.544	0.587
	*R*^2^ = 0.05, *MSE* = 3.58				
	*F*(5, 398) = 3.76, *p* < 0.01				

**FIGURE 4:**
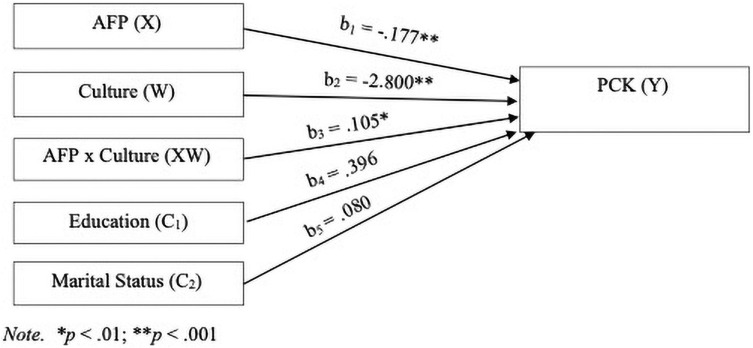
Main and interaction effects of authoritarian filial piety (AFP) and culture on palliative care knowledge (PCK). ^∗^*p* < 0.01; ^∗∗^*p* < 0.001.

**FIGURE 5:**
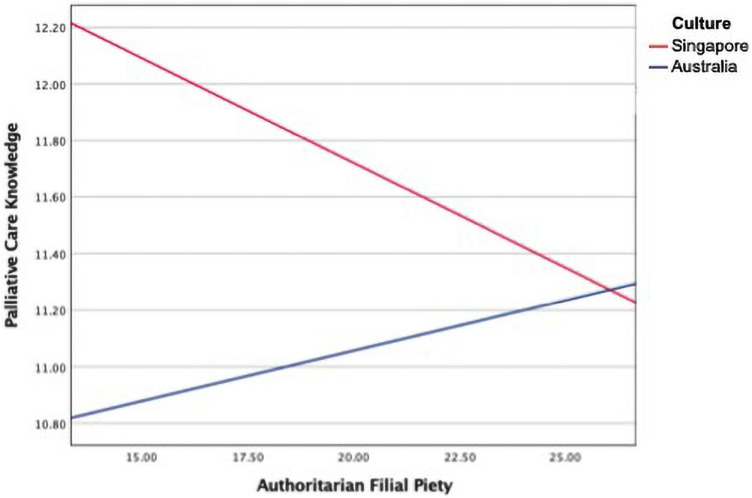
The moderating effect of culture on the relationship between authoritarian filial piety and palliative care knowledge.

The moderation analysis suggested that the moderation model explained a statistically significant 6.2% of the variation in palliative care knowledge, *F*(1, 398) = 10.60, *p* < 0.01. Authoritarian filial piety was a significant negative predictor of palliative care knowledge whilst controlling for education and marital status in the model, *b* = −0.177, *t*(398) = −3.57, *p* < 0.001, bias-corrected 95% CI [−0.275, −0.080]. Culture was also a significant predictor of palliative care knowledge whilst controlling for education and marital status in the model, *b* = −2.80, *t*(398) = −4.03, *p* < 0.001, bias-corrected 95% CI [−4.165, −1.435]. Figures 4, 5 and Table 5 display the regression parameter estimates, statistical diagram, and visual representation of the moderation model, respectively. Hence, the analysis provided partial affirmation for RQ4.

**TABLE 5:** Summary of regression analysis examining the moderation effect of culture on the relationship between authoritarian filial piety and palliative care knowledge.

		Coeff.	*SE*	*t*	*p*
Constant	*i*_*Y*_	15.106	1.148	13.162	<0.001
Authoritarian filial piety (*X*)	*b*_1_	−0.177	0.050	−3.574	<0.001
Culture (*W*)	*b*_2_	−2.800	0.695	−4.032	<0.001
Authoritarian filial piety × culture (*XW*)	*b*_3_	0.105	0.032	3.256	<0.01
Education (*C*_1_)	*b*_4_	0.337	0.209	1.612	0.108
Marital status (*C*_2_)	*b*_5_	0.107	0.111	0.964	0.336
	*R*^2^ = 0.06, *MSE* = 3.52				
	*F*(5, 398) = 5.24, *p* < 0.001				

## Discussion

### Differences in Filial Piety Between the Singapore and Australia Samples

The analysis of RQ1 indicates no significant differences in the mean score of reciprocal filial piety between the two cultural groups. This finding is interesting, as it suggests that reciprocal filial piety is not only valued but also practiced in Singapore and Australia in a similar fashion. Evidently, the concept of reciprocal filial piety, although derived from Confucianism, underlies love and care for one’s parents across the two cultures similarly. Ontologically, Confucianism regards a person’s life as an extension of their parents’ physical lives, and thus a person exists only because their parents give them their life. Consequently, the greatest gift a person receives from their parents is life itself (Sung, 1995; Li et al., 2021b). Therefore, their sincere support and grateful attitudes to their parents manifested in reciprocal filial piety. Although ontologically, the Christian view advocates a transcendent creator for human beings, reciprocal filial piety echoes the Western concept of social reciprocity that is a behavioral response to reciprocate the kindness received from other people (Falk and Fischbacher, 2006). In a positive parent-child relationship, the Western concept of reciprocity suggests a mutual exchange of supportive actions, materials, energy, time, and emotion between the parent and child, consistent with the reciprocal filial piety measured by the dual filial piety scale. As such, it is not surprising that there were no significant differences in reciprocal filial piety between the Singaporean and Australian participants.

Authoritarian filial piety is derived from the Confucian belief of absolute parental authority over the child and undisputed blind obedience to the parent (Lieber et al., 2004). This belief is reflective in the authoritarian filial piety subscale that includes question such as “Give up my aspirations to meet my parents’ expectations,” “Take my parents’ suggestions even when I do not agree with them,” and “Avoid getting married to someone, my parents dislike.” In contrast to this hierarchical parent-child relationship derived from Confucius’ authoritarian father-son relationship (Hwang, 2001; Li et al., 2021c), Australians who live in a democratic society are likely to favor individual freedom, personal autonomy, and independence in the process of decision making (Stroup, 2007) over the authoritarian parent-child relationship. As a result, Australian participants in the current study scored significantly lower in authoritarian filial piety than their Singaporean counterparts.

### Differences in Palliative Care Knowledge Between the Singapore and Australia Samples

In terms of RQ2, the Singaporean participants scored significantly higher in palliative care knowledge than their Australian counterparts. Australia was ranked at second place in the 2015 Quality of Death Index (The Economist Intelligence Unit [EIU], 2015), which was 10 places higher than Singapore. Australia also has a higher palliative care utilization rate compared to Singapore. It was thus expected that Australians would have higher levels of palliative care knowledge. According to the Singapore Department of Statistics (2021), Singapore’s population is aging. The percentage of Singaporean citizen aged 65 years and above is projected to increase from 14.4 percent in 2017 to 19 percent in 2030 (Australian Trade and Investment Commission, 2021). The proportion of Australians aged 65 years and over is projected to grow from 15 percent in 2017 (Australian Institute of Health and Welfare [AIHW], 2018) to 16.6 percent in 2030 (Commonwealth of Australia, 2010). Hence, the Singapore population is projected to age faster than the Australian population. This demographic shift places additional pressure on Singaporeans to provide old-age support to the elderly, both financially and health-wise. To tackle the palliative care pressure, numerous community initiatives and research on death and dying in Singapore have been established to promote good death (Lien Centre for Palliative Care, 2011). The initiatives and studies strongly promote palliative care literacy and awareness regarding end-of-life care options. For example, according to the report entitled *Leaving Well: End-of-Life Care Policies in Singapore*, most Singaporeans want a “good death” (Arivalagan and Gee, 2019). These initiatives may successfully promote palliative care as a public health issue. Consequently, Singaporeans develop higher levels of public awareness and knowledge of palliative care, which may contribute to the higher scores in palliative care knowledge more in Singaporean than Australian participants.

### The Relationship Between Filial Piety and Palliative Care Knowledge

The findings of the present study provide partial affirmation for RQ3. Overall and in the Australian samples, both reciprocal and authoritarian filial piety was not associated with palliative care knowledge. In the Singaporean sample, authoritarian filial piety was negatively correlated with palliative care knowledge with small effect size, while reciprocal filial piety was not associated with palliative care knowledge.

Several factors may explain the finding that filial piety (both reciprocal and authoritarian) did not correlate with palliative care knowledge. First, according to the contextualized personality theory (Heller et al., 2007; Dunlop, 2015; Dunlop and Hanley, 2019), different roles (e.g., an adult child and a caregiver) can activate different cognitive-affective mental representations and different associated meanings, people thus act differently in different roles (Fisher et al., 2017). For instance, as children, people sometimes become preoccupied with the fear of their parent’s death. To overcome the fear, they may avoid anything related to the topic of death, including learning about palliative care. As caregivers, people may be motivated to learn more about palliative care as a result of the promotion of palliative care services (in both Singapore and Australia). However, at a given moment in time, only a subset of these two role-identities is cognitively activated and accessible (Heller et al., 2009). In the context of the current study, the child role identity may be activated and more accessible than the caregiver identity. The child role-identity may evoke participants’ fear of their parents’ death, and result in their avoidance behavior toward palliative care knowledge.

Second, the inconsistency between attitude and behavior may be a contributing factor. The filial piety scale measures people’s attitudes toward their parents (Yeh and Bedford, 2003). Existing literature has suggested that if people behave in a certain way, they often have a positive attitude toward the behavior (Gilovich et al., 2019). No relationship between palliative care knowledge and filial piety is indicative that while positive filial affect exists toward taking care of parents among the participants, the participants may not have a positive attitude toward palliative care, which may hinder them from acquiring palliative care knowledge. For instance, palliative care may promote people to think about filial bereavement, which is likely to have a negative impact on people in the form of psychosomatic discomfort and emotional upset (Moss et al., 1997) and even undermine people’s psychological functioning (Marks et al., 2007). Consequently, filial attitudes toward one’s parent do not facilitate the participants’ acquiring palliative care knowledge.

The third contributing factor may be the age of the participants. The average age of the overall sample was 27.27 years, which suggests that averagely the participants were born about 28 years ago in the early 1990s. In the early 1990s, Australian women most commonly had their first child in their early to mid-20s (Australian Institute of Family Studies, 2021). The information of the average age of Singaporean women having the first child in the early 1990s is not available, while the median age of Singaporean mothers at first birth was 28.6 years in 2000 (Strategy Group Singapore, 2021). These statistics suggest that the average age of the parents of the participants in the current study is likely to be under 60 years. Generally speaking, the parents of the participants with such a young age are less likely to need palliative care services. Thus, the participants with high filial piety scores may not be significantly more motivated to seek palliative care knowledge than their counterparts with low filial piety scores.

The finding that authoritarian filial piety was negatively associated with palliative care knowledge among the Singaporean participants suggested that authoritarian filial piety appears to prevent this group of people from acquiring palliative care knowledge. As discussed previously, authoritarian filial piety advocates compliance to social obligations to the parent (Yeh, 2009; Bedford and Yeh, 2019). Providing a seriously ill parent with palliative care—indicating the cessation of life-prolonging treatment—may result in the parents feeling abandoned by their children, which is not socially accepted. Therefore, the higher level of authoritarian filial piety often brings about a greater level of social compliance, which in turn may lead to a lower level of the acquisition of palliative care knowledge.

### Moderating Effect of Culture on the Relationship Between Filial Piety and Palliative Care Knowledge

The results of the current study provide a partially affirmative test for RQ4. No interaction was found between culture and reciprocal filial piety in the moderation analysis. This finding suggests that the positive correlation patterns between reciprocal filial piety and palliative care knowledge were similar in the two cultural groups. In other words, the lack of interaction between culture and reciprocal filial piety variables in predicting palliative care knowledge indicates that the patterns of the relationship between reciprocal filial piety and palliative care knowledge were the same across the two cultural groups; that is, the higher levels of reciprocal filial piety, the higher levels of palliative care knowledge.

Culture moderated the relationship between authoritarian filial piety and palliative care knowledge. High authoritarian filial piety was associated with increased palliative care knowledge among Australians, while high authoritarian filial piety was associated with decreased palliative care knowledge among Singaporeans. That is, the direction of association relations between authoritarian filial piety and the level of palliative care knowledge depends on culture.

### Filial Piety as a Universal Construct Across Singaporean and Australian Cultures

To achieve the secondary research aim of the present study—examining whether filial piety is a universal construct across the two cultural groups—it is critical to first test the measurement invariance of the dual filial piety scale. The data of the present study supported configural invariance, metric invariance (or weak invariance) and partial scalar invariance (or partial strong invariance) of the dual filial piety scale across the Singaporean and Australian groups. According to Kline (2016), configural invariance indicates that participants across two cultural groups conceptualize the construct of filial piety in the same way. The support of metric invariance suggests that filial piety is manifested in the same way across the two groups; specifically, the slopes of regressing the items on reciprocal and authoritarian filial piety are equal across groups. Partial scalar invariance demonstrates partial equality of intercepts, suggesting that Singaporean and Australian participants in the current study used the response scale (namely, the Likert scale) of 10 items (among the 16 items, the intercept restrictions of 6 items were freed) in the same way. In other words, a person from Singapore and a person from Australia with the same level of filial piety should obtain the same score of those 10 items. As a result of the achievement of measurement invariance, the critical assumption, in this cross-cultural comparative research that the filial piety scale measures the same constructs of reciprocal and authoritarian filial piety in the same way across the two cultural groups, is met (Byrne and Van de Vijver, 2010).

Although the finding that reciprocal filial piety did not differ in the two cultural groups (see the section “Differences in Palliative Care Knowledge Between the Singapore and Australia Samples”) suggests that reciprocal filial piety is cognitively available to the participants, the current study lacks evidence that reciprocal filial piety has the same use and is accessible to the same degree in the two cultures. Thus, it is difficult to claim that reciprocal filial piety is an accessibility universal (Norenzayan and Heine, 2005). The lack of interaction between culture and reciprocal filial piety in predicting palliative care knowledge shows that the patterns of the relationship between reciprocal filial piety and palliative care knowledge were the same, although the strength of the pattern differed in the two cultural groups (as shown in Figure 3). This finding suggests that reciprocal filial piety may be a functional universal (Norenzayan and Heine, 2005) shared by the Singaporean and Australian participants in the current study.

The finding that the mean scores of authoritarian filial piety were significantly different between the two cultural groups (see the section “Differences in Palliative Care Knowledge Between the Singapore and Australia Samples”) indicates that authoritarian filial piety is differently accessible across cultures. Thus, authoritarian filial piety is not an accessibility universal (Norenzayan and Heine, 2005). As a result of the moderating effect of culture, the relationship between authoritarian filial piety and palliative care knowledge across the two cultures were in different directions (as shown in Figure 5), meaning that authoritarian filial piety fails the test of a functional universal due to the qualitatively distinct patterns emerging across the two cultures (Norenzayan and Heine, 2005). In other words, authoritarian filial piety is not a functional universal. Despite these cultural variations, the tests of measurement invariance and findings of RQs 1, 3, and 4 suggests that authoritarian filial piety not only cognitively exists in Singaporean but also in Australian cultures, albeit in significantly different degrees. As such, authoritarian filial piety appears to be an existential universal across the two cultures.

In summary, although reciprocal and authoritarian filial piety were at different levels of universals (e.g., functional and existential universals, respectively), empirical support is offered to the claim of generalizability of filial piety across Singaporean and Australian cultures within the context of the current study. It is pertinent to point out that the generalizability of filial piety is encouraging but not conclusive. More evidence needs to be sought from samples beyond the two cultures and variables in the current studies.

### Limitations and Future Directions

There are several limitations of this study that should be noted. First, Malay participants only consisted of 3.6% of the Singaporean sample. Considering that the Malay population is the second largest population group in Singapore, the generalization of the findings of this study needs to be exerted with caution due to the limitation in the relevant representative sample. Second, the current study is limited to two cultures. As pointed out by Boer et al. (2018), comparison using two cultures may lead to the interpretation paradox that differences between the two cultures which differ in terms of social, economic, and cultural factors can be easily detected, but the interpretation of the found differences and what factors explain those differences are hard to determine because the “cultural differences” may be caused by any other varying characteristics of the samples. This limitation warrants future studies that recruit participants from more cultures, particularly from China where filial piety originated. Third, given the theoretical emphasis on the ideological embeddedness of the concepts of *Xiao, seva*, and *Ketaatan Kepada Ibu Bapa*, this study would have been strengthened if the participants’ adherence to Confucian, Hindu, Malay, or Christian beliefs, and the dimensions of spirituality emphasized by Buddhism and other religions, would have been included in the study. Fourth, it is notable that the reliability coefficient of the palliative care knowledge scale was under0.70. This may be caused by the binary format of the scale. The binary answers may result in the loss of information due to the reduction of response chance (Grassi et al., 2007), which may lead to the low levels of the reliability coefficient. Future research into performance comparison of binary and Likert formats is warranted. Fifth, although this paper discussed that age may be a contribution factor to the lack of association between filial piety and palliative care knowledge (see the section “Differences in Palliative Care Knowledge Between the Singapore and Australia Samples”), the impact of age on this relationship did not test due to the research focus indicated in the RQs. Similarly, other sociodemographic factors were not included in the moderation models (while education and marital status were entered into the models as covariates). This limitation warrants future studies to extend the moderation models to include age and sociodemographic factors as moderators because these factors may a deeper and more plural understanding of the relationship between filial piety and palliative care knowledge. It is possible that for samples with ages and sociodemographic factors different from the current study, palliative care knowledge may not be relevant to assess filial piety as a functional universal. Sixth, the relationship between palliative care knowledge and palliative care utilization was not investigated in the current study. Although Li et al.’s (2021a) systematic review concludes that the lack of palliative care knowledge contributes to low levels of palliative care utilization, the current study does not appear to support this conclusion. If the findings of the current study could be translated into Singapore’s population, compared to Australia, the higher level of palliative care knowledge among Singaporean seemingly does not lead to a higher level of utilizing palliative care services. Future studies on the relationship between palliative care knowledge and palliative care utilization are needed.

## Conclusion and Implications

In conclusion, the current study indicates that the mean scores of reciprocal filial piety did not differ, but authoritarian filial piety differed across the two cultures. The levels of palliative care knowledge among Singaporeans were significantly higher than Australians. Reciprocal filial piety was not associated with palliative care knowledge in the overall Singaporean and Australian samples. Authoritarian filial piety was not associated with palliative care knowledge in the overall and Australian samples but was negatively correlated with palliative care knowledge in the Singaporean sample. Culture did not moderate the relationship between reciprocal filial piety and palliative care knowledge but moderated the relationship between authoritarian filial piety and palliative care knowledge. Reciprocal and authoritarian filial piety appear to be a functional and an existential universal, respectively, across the two cultures.

To the authors’ knowledge, this is the first study to provide empirical evidence that filial piety demonstrates generalizability across Singaporean and Australian cultures within the context of global aging. The findings have theoretical, empirical, and practical implications. Theoretically, the present findings support the notion that the conceptualization of filial piety can be moved beyond the traditional notion that regards filial piety as a cultural norm to an innovative point of view that considers filial piety as a possible psychological universal. This move is particularly welcome as an example of cross-cultural research originating from a non-Western cultural concept—“filial piety.” Cross-cultural research has been long conducted from the perspective of a Western concept with a tendency to construe divergences from the Western concept to conclude a cultural deviation or deficit in non-Western societies. Empirically, through the analysis of measurement invariance and the data, the current study validates the cross-cultural applicability of the dual filial piety model, which provides empirical support to the employment of filial piety as a contextualized personality construct in the future research on intergenerational relations in Western societies. The testing of measurement invariance also adds value to Norenzayan and Heine’s (2005) three-step approach to probing universal constructs in cross-cultural research in that measurement invariance is largely overlooked in Norenzayan and Heine’s approach. Practically, given that the present study finds that higher levels of authoritarian filial piety were associated with decreased palliative care knowledge among Singaporeans, public health palliative care in Singapore should consider addressing this relationship. Considering that higher levels of authoritarian filial piety were associated with increased palliative care knowledge in Australians, public health palliative care could utilize authoritarian filial piety to enhance palliative care knowledge among Australians. Integrating filial piety into the public health approach to palliative care may also benefit patients, caregivers, and healthcare provision in the COVID-19 pandemic.

## Data Availability Statement

The datasets generated for this study can be found in Research Data JCU: https://doi.org/10.25903/5fdw-ne77.

## Ethics Statement

The studies involving human participants were reviewed and approved by the Human Research Ethics Committee James Cook University, Australia (Ref. H8021). The patients/participants provided their written informed consent to participate in this study.

## Author Contributions

WL directed the project and significantly contributed to the conceptualization of the study, design of the data analysis, and writing the major portion of the manuscript. SS considerably contributed to the research design, data collection, data analysis, drafting the manuscript, and critically reviewing the manuscript. CK considerably contributed to the acquisition of data, data analysis and interpretation, drafting the manuscript, and critically reviewing the manuscript. All authors reviewed the results and approved the final version of the manuscript.

## Conflict of Interest

The authors declare that the research was conducted in the absence of any commercial or financial relationships that could be construed as a potential conflict of interest.

## Publisher’s Note

All claims expressed in this article are solely those of the authors and do not necessarily represent those of their affiliated organizations, or those of the publisher, the editors and the reviewers. Any product that may be evaluated in this article, or claim that may be made by its manufacturer, is not guaranteed or endorsed by the publisher.
